# The injury mortality burden in Guinea

**DOI:** 10.1186/1471-2458-12-733

**Published:** 2012-09-02

**Authors:** Keita Mamady, Hongyan Yao, Xujun Zhang, Huiyun Xiang, Hongzhuan Tan, Guoqing Hu

**Affiliations:** 1 Department of Epidemiology and Health Statistics, School of Public Health, Central South University, 110 Xiangya Road, Changsha, 410078, China; 2 Epidemiology Office, Chinese Center for Disease Control and Prevention, Beijing, China; 3 School of Public Health, Southeast University, Nanjing, China; 4 Center for Injury Research and Policy, The Research Institute at Nationwide Children’s Hospital, The Ohio State University, Columbus, USA

**Keywords:** Injury, Mortality, Cause of death, Guinea

## Abstract

**Background:**

The injury mortality burden of Guinea has been rarely addressed. The paper aimed to report patterns of injury mortality burden in Guinea.

**Methods:**

We retrieved the mortality data from the Guinean Annual Health Statistics Report 2007. The information about underlying cause of deaths was collected based on Guinean hospital discharge data, Hospital Mortuary and City Council Mortuary data. The causes of death are coded in the 9^th^ International Classification of Diseases (ICD-9). Multivariate Poisson regression was used to test the impacts of sex and age on mortality rates. The statistical analyses were performed using Stata^tm^ 10.0.

**Results:**

In 2007, 7066 persons were reported dying of injuries in Guinea (mortality: 72.8 per 100,000 population). Transportation, fire/burn, falls, homicide and drowning were the five leading causes of fatal injuries for the whole population, accounting for 37%, 22%, 12%, 10% and 6% of total deaths, respectively. In general, age-specific injury causes displayed similar patterns of the whole population except that poisoning replaced falls as a leading cause among children under five years old. Males were at 30-50% more risk of dying from six commonest causes than females and old age groups had higher injury mortality rates than younger age groups.

**Conclusion:**

Transportation, fire/burn, falls, homicide, and drowning accounted for the majority of total injury mortality burden in Guinea. Males and old adults were high-risk population of fatal injuries and should be targeted by injury prevention. Lots of work is needed to improve weak capacities for injury control in order to reduce the injury mortality burden.

## Background

Violence and injuries are a major global public health issue, accounting for more than 5 million deaths every year -- as many deaths as from HIV, malaria and tuberculosis combined [1]. In Guinea, injury was the fifth leading cause of morbidity burden in 2008 [2]. Despite the huge loss due to injuries, injury control receives little attention of policy-makers and researchers in this country. As well as in most developing countries, research on injury control has been severely under-funded compared with infectious diseases in Guinea [3]. Consequently, few peer-reviewed publications addressed the injury problem in Guinea.

The only accessible information source about Guinea’s injury burden is the country-specific disease burden estimate from *The Global Burden of Disease: 2004 Update*[4]. The global burden of disease study used the cause-of-death model (a formalized simple spreadsheet program) to estimate the disease burden of Guinea based on the data from South African death registration data 2004, Zimbabwe National Burden of Disease Study 1997, INDEPTH verbal autopsy data from 7 sites in Africa 1999–2002, Antananarivo Madagascar 1976–1995, and Mozambique Maputo Central Hospital Mortuary 1993–2004 [4]. In fact, the injury burden varies greatly across countries. Peltzer observed large variations in the prevalence of serious injury between six African countries and suggested that each country document its epidemiology of serious injuries [5]. The estimates of injury burden based on other countries’ data may not reflect the reality in Guinea.

The Ministry of Public Health of Guinea releases the Guinean Annual Health Statistics Report annually, which is based on the Guinean Hospital Discharge Registry, Hospital Mortuary and City Council Mortuary data [3]. Unfortunately, the Guinean Annual Health Statistics Report has rarely been used for injury control and research purposes because of the technical and language limitations. This present study aimed to report the latest injury mortality burden of Guinea based on the Guinean Annual Health Statistics Report 2007, thus increasing the knowledge of injury epidemiology in Africa.

## Methods

### Data source

Mortality data were obtained from the Guinean Annual Health Statistics Report 2007. The Ministry of Public Health of Guinea makes this report accessible to the public and a hard or electronic copy could be requested by contacting the assigned officials ( http://www.stat-guinee.org/nada/index.php/catalog/1/accesspolicy/). The data were collected based on Guinean Hospital Discharge Register, Hospital Mortuary and City Council Mortuary data. In Guinea, the national health system is a pyramid of facilities, comprising two national hospitals (teaching hospitals), seven regional hospitals, 26 prefectural hospitals, 8 communal medical centers, 390 health centers, and 628 health posts [3]. Guinea adopts standard clinical and laboratory methods to diagnose the cause of diseases under the support from World Health Organization (WHO) and other partners of development [6]. The causes of death are coded using the 9^th^ International Classification of Diseases (ICD-9). The national public health laboratory of Guinea regularly inspects all the laboratories and holds up-to-date training to laboratory assistants at local levels [3].

The Guinean Hospital Discharge Register collects the data of patients who die at hospital ward based on discharge records. The Guinean Hospital Discharge Register is a compilation of discharge records of all hospitals which are collected at regional level and then are reported to the Ministry of Public Health by local health administrative authorities [6].

For patients who die on arrival, the bodies of patients are temporarily kept in hospital mortuary, where doctors and trained nurses record the information on demographics, date and mechanism of deaths of patients through the interview with the accompanying persons. Hospital mortuary data are collected locally at each hospital by the physicians and then reported to the Board of Health where an intensive review will be conducted to check the data [3].

City Council Mortuary keeps the records of persons who die out of hospital including the information on demographics, date of death, last known address of victim and the detail autopsy findings [3]. The Ministry of Health directly collects post-mortem reports from City Council Mortuary.

The Ministry of Health merged three kinds of data sources above and removed duplicates to produce official health statistics. With the assistance from WHO, the proportion of death reporting reached 99.7% at local health departments and 100% in hospitals [3].

Guinea’s population was estimated to be 9710144 inhabitants in 2007, on the basis of estimates of the 1996 census [3].

### Statistical analysis

Mortality rates were used to measure the burden of fatal injury, calculated as the number of deaths divided by the population size × 100,000. The age was divided into five groups: 0–4 years, 5–14 years, 15–24 years, 25–64 years and 65 years and over. The causes of injuries included five unintentional categories (drowning, fire/burn, transportation, falls, and poisoning), two intentional categories (homicide and suicide), and others (the rest of causes).

The relative contributions of seven commonest external causes were displayed using percentage bar chart by age group. Multivariate Poisson regression was used to test the significance of differences from sex and age group. We used mortality rate ratio (MRR) to quantify sex-specific and age-specific differences. The statistical analyses were carried out using Stata^tm^ 10.0, and a *p* value <0.05 was selected as the statistical significance level.

## Results

In total, 7,066 persons suffered fatal injuries in Guinea in 2007 (mortality: 72.8 per 100,000 population) (Table 1). In comparison to females, males had 1.3 times risk dying of injuries (83.6 vs. 62.4 per 100,000 population). Notably, the injury mortality rate increased significantly as people got older. Children under five years old had the lowest rate (7.9 per 100,000 population) while adults ages 65 years and older had the highest rate (137.6 per 100,000 population).

**Table 1:** Mortality from injuries in Guinea, 2007

**Demographics**	**N. of deaths (%)**	**Mortality /100,000 population**	**Mortality rate ratio (95% CI)**
Total	7066 (100%)	72.8	
Sex			
Male	3963 (56.1%)	83.6	1.3 (1.3, 1.4)^*^
Female	3103 (43.9%)	62.4	Reference
Age group			
0-4	136 (1.9%)	7.9	Reference
5-14	836 (11.8%)	31.1	3.9 (3.3, 4.7)^*^
15-24	1620 (22.9%)	101.5	12.8 (10.8, 15.3)^*^
25-64	3824 (54.1%)	118.5	14.9 (12.6, 17.7)^*^
65+	650 (9.2%)	137.6	17.4 (14.5, 20.9)^*^
Intention/cause			
Drowning	430 (6.1%)	4.4	
Fire/burn	1519 (21.5%)	15.6	
Transportation	2644 (37.4%)	27.2	
Falls	829 (11.7%)	8.5	
Poisoning	252 (3.6%)	2.6	
Homicide	679 (9.6%)	7.0	
Suicide	11 (0.2%)	0.1	
Others	702 (9.9%)	7.2	

*: *p* < 0.05.

For the whole population, transportation, fire/burn, falls, homicide and drowning were the five leading causes of injury-induced deaths, accounting for 37%, 22%, 12%, 10% and 6% of total injury-related deaths, respectively (Table 1). Relative to the 7.0 per 100,000 population for homicide rate, the suicide rate was extremely low -- 0.1 per 100,000 population.

**Figure 1:**
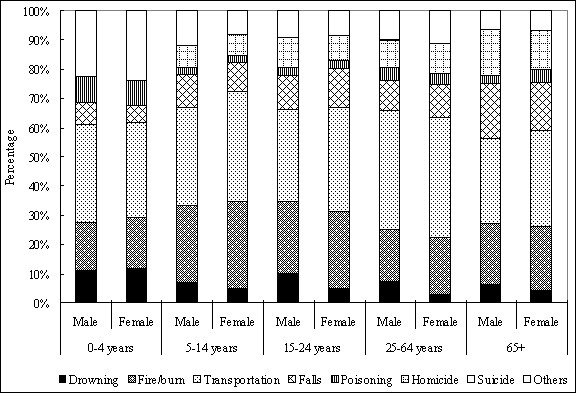
Percentage of death causes of injuries by age group (Guinea, 2007).

In general, persons aged five years and older displayed similar injury cause patterns for both sexes as the whole population; whereas among children under five years old, poisoning replaced falls as a common cause (Figure 1). Compared with females, males showed a similar injury-cause pattern except that drowning contributed a little more in males than in females for the latter four age groups (age ≥5 years old) (Figure 1).

Males were at 30-50% more risk of dying from fire/burn, transportation, falls, poisoning and homicide than females (Table 2). The male/female difference was 1.8 times for drowning mortality. After adjusting for sex, older age groups had higher mortality rates than children under-five years old for drowning, fire/burn, transportation, falls, and positioning. The homicide mortality significantly increased over age among persons aged 5 years and older.

**Table 2:** Mortality rate ratio (95% confidence interval) of injury mortality by leading causes (Guinea, 2007)

**Sex/age group**	**Drowning**	**Fire/burn**	**Transportation**	**Falls**	**Poisoning**	**Homicide**
Sex						
Male	2.8 (2.3, 3.5)^*^	1.3 (1.2, 1.5)^*^	1.3 (1.2, 1.5)^*^	1.4 (1.2, 1.6)^*^	1.4 (1.1, 1.8)^*^	1.5 (1.2, 1.7)^*^
Female (reference)						
Age group						
0-4 years (reference)						
5-14 years	2.0 (1.1, 3.5)^*^	6.5 (4.2, 9.9)^*^	4.2 (3.1, 5.8)^*^	6.3 (3.2, 12.5)^*^	1.1 (0.5, 2.2)	reference^†^
15-24 years	8.9 (5.3, 15.0)^*^	19.5 (12.8, 29.7)^*^	13.0 (9.6, 17.6)^*^	24.7 (12.7, 48.2)^*^	3.6 (1.9, 6.9)^*^	4.4 (3.3, 6.0)^*^
25-64 years	7.0 (4.2, 11.7)^*^	16.6 (11.0, 25.2)^*^	18.8 (14.0, 25.2)^*^	24.7 (12.8, 47.8)^*^	7.1 (4.0, 12.8)^*^	5.1 (3.9, 6.7)^*^
65+	7.9 (4.4, 14.3)^*^	22.5 (14.5, 34.9)^*^	16.3 (11.8, 22.5)^*^	46.9 (23.8, 92.4)^*^	6.7 (3.3, 13.6)^*^	9.1 (6.6, 12.5)^*^

*: *p* < 0.05.

†: Mortality rate ratio for age group was calculated using the reference age group (15–24 years) because of the lack of deaths in the youngest age group.

Note: Mortality rate ratios for suicide were omitted due to extremely low age-specific suicide rate and the absence of deaths in the reference groups.

## Discussions

More than 7,000 persons died of injuries in Guinea in 2007. Males were 30% more likely to experience a fatal injury than females. The overall injury risk increased as people got older. Transportation, fire/burn, falls, homicide, and drowning were the five leading causes of injuries for all ages combined, accounting for 87% of all fatal injuries. An exception was the poisoning as the fourth leading cause of death for children less than five years old.

**Figure 2:**
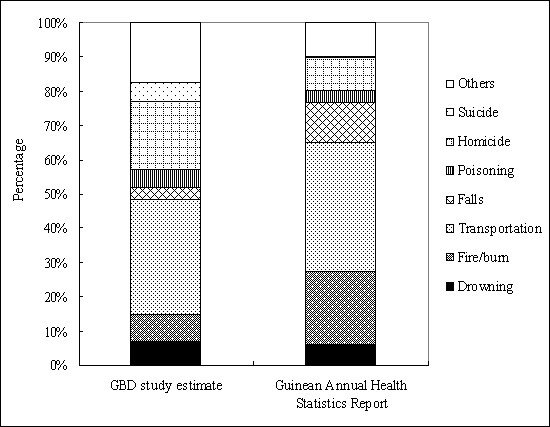
Comparison of injury cause composition between global burden of disease (GBD) study estimate and Guinea’s statistics.

Compared to the estimates from *the Global Burden of Disease: 2004 Update* for Guinea (86.9 per 100,000 population) [4], the rate based on Guinea’s official statistics (72.8 per 100,000 population) is lower. More importantly, the death cause patterns of injuries obviously differ between two data sources (Figure 2). For example, the percentages of fire/burn, falls, homicide, and suicide were 7.9%, 3.2%, 19.9%, 5.8%, respectively, based on the estimates of GBD 2004 update; [4] while according to Guinea’s official statistics, they were 21.5%, 11.7%, 9.6%, and 0.2%. The observed discrepancy indicates large variations in the injury death patterns across countries in Africa, stressing that the policy-making for injury control should be based on own data for each country.

As well as in most countries, transportation was the most important cause of injury-induced deaths in Guinea. The large transportation-related deaths may be due to increasing motorization, inadequate adoption and enforcement of traffic laws, high traffic law violation, poor traffic control, and lack of adult supervision of children [7-9]. Six recommendations proposed by the *World Report on Road Traffic Injury Prevention*[10], have the potential to be used to improve the transportation safety in Guinea, including 1) identify a lead agency in government to guide the national road traffic safety effort; 2) assess the problem, policies and institutional settings relating to road traffic injury and the capacity for road traffic injury prevention; 3) prepare a national road safety strategy and plan of action; 4) allocate financial and human resources to address the problem; 5) implement specific actions to prevent road traffic crashes, minimize injuries and their consequences and evaluate the impact of these actions; and 6) support the development of national capacity and international cooperation.

Fire/burn constituted more than 20% of total injury-induced deaths in Guinea, which differed from in other African countries [4]. The high prevalence of fire/burn-induced deaths could be ascribed to unsafe power sources for cooking, lighting, and heating, crowding and flammable house [11-14]. In Guinea, a typical house is a one-room with two doors but without windows [15]. The room is made of mud walls and a roof of bamboo and leaves [15]. One hut is usually crammed with four or more persons to sleep [15]. These factors make house fire a significant contributor to fire-related deaths in Guinea. Improvement in the housing system and introduction of safe cooking, lighting and heating, may have the potential to decrease unnecessary fire-induced fatalities.

It is not unique for Guinea that males and old adults are high-risk population for fatal falls. However, there is a lack of scientific data in support of development of fall prevention programs because little is known about the mechanism of falls for Guineans, like personal behaviors, environmental factors, engineering factors. Clearly, further studies are needed in Guinea to identify protective and risk factors of falls, to develop and evaluate fall prevention programs. In general, a multidisciplinary approach is needed but environmental modifications should be emphasized [16]. In the last several decades, many prevention programs have been developed to decrease fall-induced injuries in older population in the developed countries [17]. Guinea could benefit from successful interventions from other countries but needs to consider necessary modifications when introducing them because of huge cultural differences between Guinea and these countries.

Violence, particularly homicides, has become a major public concern for Guinean society. The result may be due to primarily social instability from frequently alternated government and lack of enforcement of laws [18-20]. Although the awareness campaigns against violence have been launched by the government, international organizations and other partners recently [21], more actions are needed. Especially, strictly-designed studies should be conducted to develop cost-effective interventions to prevent violence.

The extremely low suicide rate may be partially due to the impact of religious belief of Muslim. According to the official statistics, approximately 85% of Guineans (7.8 million) are Muslim [22]. The doctrines of Muslim regard suicide as one of the greatest sins and utterly detrimental to one's spiritual journey. A verse in the Quran instructs, ‘*And do not kill yourselves, surely God is most merciful to you*.’ [23] On the other hand, however, the low suicide rate may also be caused by potential under-reporting in terms of poor data quality of many developing countries [24] and social stigma associated with suicide [25].

Poisoning caused a significant proportion of child fatal injuries in Guinea. As the *World Report on Child Injury Prevention* summarizes, the greatest obstacle for world child poisoning control is the lack of reliable data [26]. Currently, there are few poisoning data collection systems but almost all of them are located in the developed countries [26]. Although measures like poison control centers, hotline, child-resistant packaging, and education (including training of parents and caregivers) combined with home visitations, have been found somewhat effective, few measures have been tested in the developing countries [25]. It is important for Guinea to develop a separate data collection system for poisoning control, or to include poisoning data needed in the existing data system. Furthermore, rigorous studies are needed to test the effectiveness of successful prevention interventions from developed countries in the local communities.

This study is primarily limited by the data quality of Guinea’s official statistics. The real injury mortality may be underestimated to some degree because some deaths that occurred out of hospital and the corpses were not sent to the mortuary might have not been counted by the official statistics. Additionally, injuries might have been misclassified as other diseases or conditions in the remote areas considering poor condition of medical care centers, lack of experienced health care providers [27,28].

## Conclusion

Transportation, fire/burn, falls, violence are the most important causes of injury-induced deaths in Guinea. Males and old age groups are at high risk of dying of injuries. The huge burden of fatal injuries in Guinea calls for a multidisciplinary and multi-sectors approach in raising awareness of and in developing cost-effective programs to prevent those devastating injuries.

## Abbreviations

HDR: Hospital discharge register; ICD-9: 9th International Classification of Diseases; WHO: World Health Organization.

## Competing interests

We declare we have no competing interests.

## Authors' contributions

Keita Mamady and Guoqing Hu designed the study, completed data analysis and drafted the paper, Hongyan Yao, Xujun Zhang, Huiyun Xiang, and Hongzhuan Tan helped interpret the results and improve the manuscript. All authors read and approved the final manuscript.

## Pre-publication history

The pre-publication history for this paper can be accessed here:

http://www.biomedcentral.com/1471-2458/12/733/prepub
